# Residual serum fibrinogen as a universal biomarker for all serotypes of Myasthenia gravis

**DOI:** 10.1038/s41598-023-47559-x

**Published:** 2023-12-01

**Authors:** Faraz S. Hussain, Ramanaguru S. Piragasam, Hassan Sarker, Derrick Blackmore, Elaine Yacyshyn, Carlos Fernandez-Patron, Richard P. Fahlman, Zaeem A. Siddiqi

**Affiliations:** 1 Division of Neurology, Department of Medicine, Faculty of Medicine & Dentistry, University of Alberta, Edmonton, Canada; 2 Department of Biochemistry, Faculty of Medicine & Dentistry, University of Alberta, Edmonton, Canada; 3 Division of Rheumatology, Department of Medicine, Faculty of Medicine & Dentistry, University of Alberta, Edmonton, Canada

**Keywords:** Neurological disorders, Proteomics, Biomarkers, Neurology

## Abstract

Myasthenia Gravis (MG) is an autoimmune disease associated with severe neuromuscular weakness. Diagnostic confirmation of MG is typically delayed and secured in about 85% and 50% of patients with generalized and ocular MG, respectively with serum antibodies. We have identified a sensitive and specific diagnostic biomarker for various MG serotypes with quantitative proteomics. Serum proteomes of 18 individuals (MG patients, healthy controls (HC), Rheumatoid Arthritis (RA) were quantified in a pilot study and occurrence of high residual fibrinogen was validated by immunoblotting and further investigated by targeted mass spectrometry on the sera of 79 individuals (31 MG of various serotypes, 30 HC, 18 RA). Initial proteomic analysis identified high residual fibrinogen in MG patient sera which was then validated by antibody-based testing. Subsequently, a blinded study of independent samples showed 100% differentiation of MG patients from controls. A final serological quantification of 14 surrogate peptides derived from α-, β-, and γ-subunits of fibrinogen in 79 individuals revealed fibrinogen to be highly specific and 100% sensitive for MG (p < 0.00001), with a remarkable average higher abundance of > 1000-fold over control groups. Our unanticipated discovery of high levels of residual serum fibrinogen in all MG patients can secure rapid bedside diagnosis of MG.

## Introduction

Myasthenia gravis (MG) is a humoral autoimmune disorder in which autoantibodies directed against neuromuscular junction (NMJ) proteins affect the electrical signal transmission across the NMJ resulting in variable weakness of voluntary muscles ranging from mild ocular and/or limb muscle weakness to fulminant life threatening myasthenic crises due to weakness of swallowing and breathing muscles^1^. As a result of its nonspecific symptoms, challenges still remain concerning diagnosis of MG in the emergency room^2,3^ and in elderly populations with comorbid illnesses^4,5^.

The prevailing thought is that autoantibodies in MG are directed against the nicotinic acetylcholine receptor (AChR) at NMJ in about 85% of patients^6^. A smaller proportion of patients have autoantibodies against other NMJ proteins, including the muscle specific tyrosine kinase (MuSK) or low-density lipoprotein receptor-related protein 4 (LRP4)^7,8^. No antibodies can be detected in about 10–15% of patients with generalized MG and in about 50% with ocular MG, using the current assays (seronegative MG) though such patients manifest clinical features and therapeutic responses similar to those with detectable autoantibodies. Although relatively specific for the diagnosis and subgrouping of MG^9^, the serum level of anti-AChR, anti-MuSK or anti-LRP4 antibodies does not correlate with the disease course or treatment outcomes. Limitations of the current molecular diagnostic methods are particularly exemplified with the number of seronegative patients, particularly those with the ocular form i.e. MG Foundation of America (MGFA) Class I, of this disease^10^.

The identification of robust serological biomarkers in MG has previously been attempted. In one study, the blood levels of proliferation-inducing ligand (APRIL) and several cytokines, such as IL-19, IL-20, IL-28A and IL-35, were found to be upregulated in the sera of MG patients as compared to controls^11^. A second study has reported correlations between MG and the serum levels of matrix metalloproteinase 10 (MMP-10), transforming growth factor alpha (TGF-α) and receptor for advanced glycation end-products binding protein (protein S100-A12)^12^. Most recently, high κ free light chain has been reported in both seropositive and seronegative forms of MG^13^.

Although these studies provide promising results, the limited dynamic range (less than twofold) of the proteins between controls and individuals with MG leads to significant challenges for their utility in robust diagnostic testing. Additionally, it is not clear whether the elevation of serum inflammatory proteins in MG patients is disease specific or represents a non-specific, general increase in the inflammatory mediators expected in an autoimmune disease. Notably, patients with MG have increased risk of having another autoimmune disorder, with about 13–22% having a second autoimmune disorder^14,15^. In line with these challenges, a recent report has identified a panel of five serum metabolites, which include three lysophospholipids, glyceric acid and 12-ketodeoxycholic acid, that are reported to differentiate between MG and another autoimmune disease Rheumatoid arthritis (RA)^16^. However, again the limited dynamic range of these metabolites, of less than twofold, impairs their utilization for diagnostic testing.

To circumvent some of the current challenges in MG diagnosis, the present study aimed to identify a serum proteomic biomarker that may be universally sensitive for all classes and serotypes of MG and exhibit specificity with regards to related diseases. To this end, our study design includes sera from a heterogeneous cohort of MG patients that was not only compared to a normal control group but also to a reference autoimmune disease, RA. The choice of RA as the reference autoimmune disease is based on the observation that like MG, RA also has a major humoral component, and the two diseases may co-exist in about 2–4% of MG patients^17,18^. We postulated that the comparison of the serological protein profiles from the two diseases and controls may potentially identify specific proteins in serum that are unique to MG.

## Materials and methods

### Ethics approval

The sera samples for the current study were obtained from Canadian BioSample Repository (CBSR), collected after obtaining informed consent and stored in a de-identified manner by Dr. Siddiqi’s MG study group. Approval to use the sera, to investigate potential biomarker(s), was obtained from the Health Research Ethics Board, University of Alberta (Ethics ID: Pro00030698). All methods were carried out in accordance with relevant guidelines and regulations while assuring the confidentiality and privacy.

### Serum samples

For the study three groups of samples were identified: MG, RA (as a reference disease), and healthy controls (HC). A total of 79 samples were recruited for the study: 31 MG, 18 RA and 30 HC. The demographics of the individuals are summarized in Table 1. MG and RA serostatus was confirmed with antibody testing for either anti-AChR/anti-MuSK (MG) or RA. The MG diagnosis for the seronegative individuals was assured through repetitive nerve stimulation studies and/or single fiber electromyography (SFEMG). All MG individuals were graded according to MGFA classification, Class I (Ocular, n = 8), Class II (n = 10) and Class III (n = 13). The diagnosis of RA was secured in accordance with the American Rheumatology Association 1987 criteria^19^. To exclude the confounds of race, only Caucasian patients were included in this study. There were no smokers and no statistically significant differences between all groups from time of last meal or BMI. Further, patients had no history of any other autoimmune disease or thymoma. Finally, due to the nature of recruitment, patients were not required to fast. During a prospective observational trial, the MG patients and healthy controls were recruited for the collection of serum samples. RA samples were collected in multiple clinics.

**Table 1:** Cohort demographics.

Myasthenia gravis	n = 31
Average age (years)	59.0 ± 23.9(range 19–93)
Gender (M/F, %)	51.6% / 48.4%
Average on-set age	54.7(range 15–86)
Early/Late on-set (%)	35.5%/64.5%
Disease severity (MGFA)	
Class I	25.8%
Class II	32.3%
Class III	41.9%
Control	n = 30
Average age (years)	48.2 ± 17.1(range 21–86)
Gender (M/F, %)	40/60
Rheumatoid arthritis	n = 18
Average age (years)	65.1 ± 11.7(range 36–81)
Gender (M/F, %)	33.3/66.7

Demographic information and clinical profiles of the three groups of individuals from which sera were collected for fibrinogen protein analysis. As per the Myasthenia Gravis Foundation of America (MGFA) classification, Class I represents purely ocular from of MG, Class II represents mild generalized MG and Class III describes moderately severe generalized MG.

For collection, blood samples were drawn from the antecubital vein using a 21 G needle and vacutainer red top no additive tubes (Becton Dickenson).

### SDS-PAGE, western blot analysis and ELISA

SDS-PAGE loading buffers contained 100 mM of β-mercaptoethanol and 4% SDS. 5 µl of samples were resolved by 10% SDS-PAGE and either visualized by Coomassie R250 staining or transferred to a nitrocellulose membrane for Western blot analysis as previously described^20^. The membranes were then immunoblotted with either a mouse anti-FGA antibody (A-6) (SC-166968; Santa Cruz Biotechnology) derived against residues 750–850 or a rabbit anti-FGA antibody (ab92572, Abcam) derived against residues 21–320. Secondary blotting was achieved with either goat anti-Mouse or donkey anti-Rabbit IRDye 680RD labeled antibodies (LI-COR).

Coomassie stained gels and Western blots were visualized with a LiCOR Odyssey Fc system.

Serum fibrinogen was quantified using an enzyme-linked immunosorbent assay (ELISA) performed with a kit (ab208036, Abcam) following the manufacturer’s recommended procedure.

### Serum protein profile by Gel-LC–MS/MS

Eighteen randomly selected samples, 6 from each group, were used for the initial shotgun proteomic analysis. Samples were resolved by 10% SDS-PAGE and visualized by Coomassie staining. Each lane was excised into 26 bands, then each band was subjected to in-gel tryptic digestion as previously described^21^. The resulting digested peptides were vacuum dried and re-suspended in solvent A (5% Acetonitrile (ACN) in 0.2% formic acid) for LC–MS/MS analysis. LC–MS/MS analysis was performed on Thermo Scientific EASY-nL 1000 system inline Q-Exactive Hybrid Quadrupole-Orbitrap Mass Spectrometer using identical parameters as previously described^22^ with the alteration of running a 75 min gradient.

### MS/MS data analysis

Raw data files of MS/MS spectra for each sample were combined as searched with Proteome Discoverer 1.4.1.14 (Thermo Fisher Scientific, US) against the non-redundant and reviewed proteome of Homo sapiens retrieved from UniProt. The search parameters were as previously described^23^. All of the identified proteins from each sample along with their associated extracted ion intensities quantified for abundance are listed in Supplementary Tables 1 and 2.

### Quantification and statistical analysis

EICs were used to quantify the protein abundance in individual samples, for which the values were subjected to normalization by the proportion to total ion current (TIC) as previously described^23^. One-way ANOVA was employed for comparative analysis of the three sample groups, using a statistical cut-off of p < 0.05. All the data used for statistical analysis is provided in Supplementary Table 3.

### Parallel reaction monitoring

Serum proteins were resolved by 8% SDS-PAGE and then prepared for LC–MS/MS as described above. The resulting fractions from each sample were then pooled prior to LC–MS/MS analysis. Multiple peptides per protein (Supplementary Table 4) were included for method building and final quantification using the Skyline software package^24^ were implemented as described previously^22^. The raw quantified data from PRM analysis is provided in Supplementary Table 5.

### Data availability and report guidelines

The MS proteomics data have been deposited to the ProteomeXchange Consortium via the PRIDE^25^ repository with the dataset identifier PXD019633. The Scientific Reporting of Proteomic Biomarker Data guidelines was used for the reporting of the study^26^.

## Results

### Myasthenia gravis serum proteome profiling

**Figure 1:**
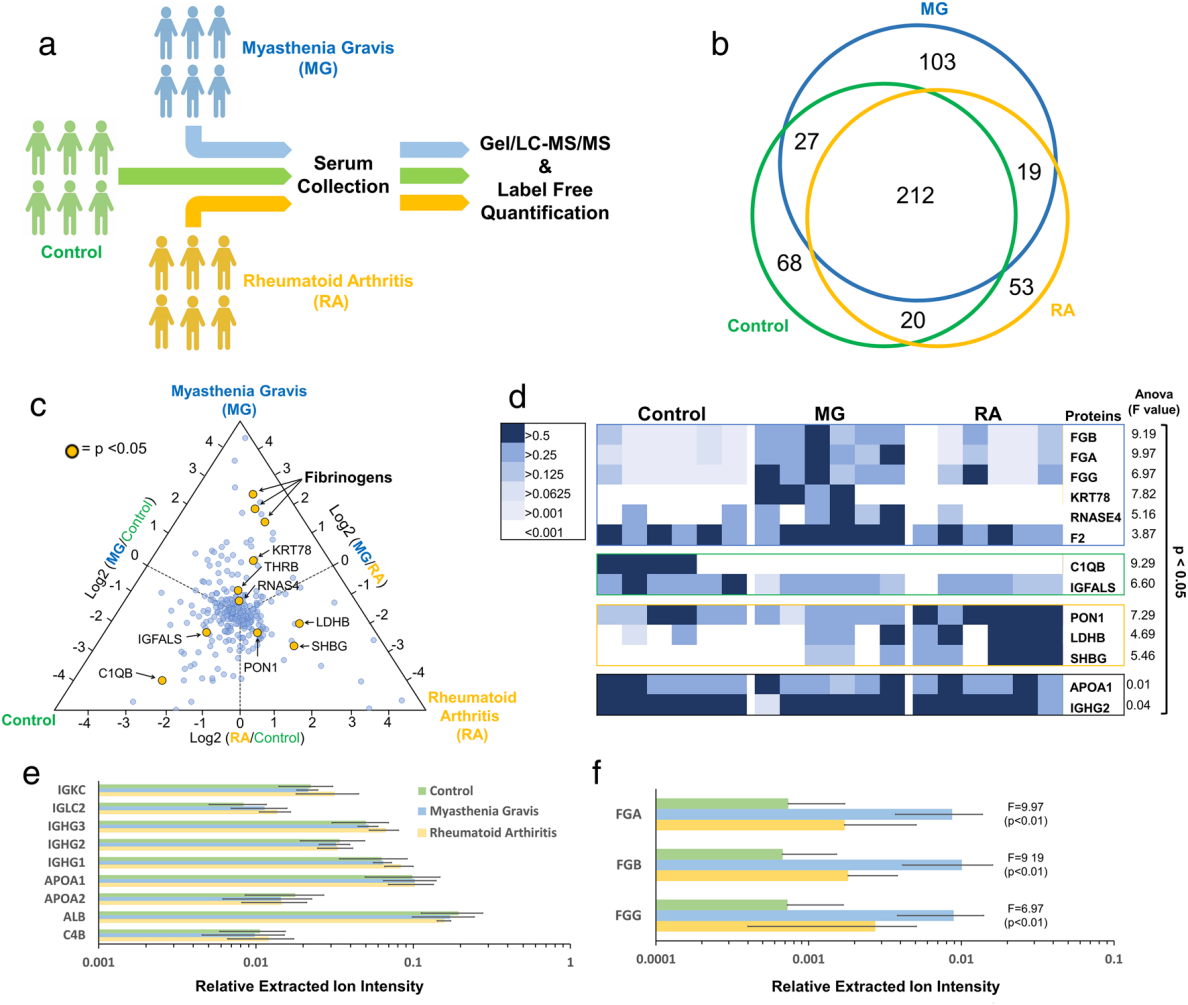
Clinical Proteomic Analysis of Myasthenia Gravis (MG) Patients. (**a**) Schematic workflow for the label free proteomic analysis of serum collected from 6 MG patients, 6 control individuals and 6 with the related disease, Rheumatoid Arthritis (RA). (**b**) Venn diagram summarizing the proteins identified and quantified among the three test groups. (**c**) A three-way triplot of differential protein abundance observed between MG, control and RA samples. The log2 ratio of the normalized protein abundance was plotted to visualize differential amounts between the three groups. In this plot, proteins observed in higher abundance in one group over others are observed closer to the respective vertices. Proteins that exhibit reproducible differences as revealed by a one-way ANOVA analysis (p < 0.05) are highlighted in yellow and labelled. (**d**) Heatmap representation of the abundance of statistically significant proteins with their respective calculated ANOVA F-statistic shown (p < 0.05). In addition, two control proteins are shown for comparison. (**e**) Validation of label-free quantification by investigating the variability of the measurements between groups for a series of serum proteins, including immunoglobulins (IGKC, IGLC2, IGHG3, IGHG2 & IGHG1); apolipoprotein A (APOA1, APOA2); serum albumin (ALB); and C4b-binding protein alpha chain (C4B). (**f**) Relative average abundance level of fibrinogen-α (FGA), fibrinogen-β (FGB) and fibrinogen-γ (FGG) among all sample groups. (Final Figures and Graphs were generated with Excel from Microsoft Office 365. Graphics were generated with Power Point from the same application package.)

To identify novel proteins in an unbiased manner that may be specifically associated with MG, a pilot study using a gel-based shotgun proteomics method (Gel-LC–MS/MS) was done for analysis of unadulterated serum samples collected from six HC, six MG patients and six RA patients, as outlined in Fig. 1a. As summarized by the Venn diagram in Fig. 1b, a total of 502 proteins were identified with the criteria of two or more quantifiable peptides per protein to limit the false discovery rate (FDR) of protein identifications. Within the MG group 361 proteins were identified, while for the HC and RA groups, 327 and 304 were identified, respectively. Of these proteins, 212 were common to all groups whereas the number of unique and additionally shared numbers of proteins for each group are indicated in Fig. 1b.

### Identification of proteins of differential abundance

The ratios of the average relative extracted ion intensity for each protein compared among all three test groups are shown in the tri-plot in Fig. 1c. The clustering of the data points in the centre of the graph reveals that the majority of average ion intensities for each protein is similar between all three groups, but that some proteins are higher in some of the groups with the data being closer to the triangle apex from the respective group. As some average ion intensities maybe biased as a result of highly variable measurements from individuals in a group, either from technical variability in quantification or true variability of a protein in an individual, all the data was analyzed using a one-way ANOVA to identify proteins that exhibit statistically significant differences between the MG, RA and HC groups. The proteins that met the criteria of a p-value < 0.05 are depicted as yellow circles in the tri-plot. While this cut-off may lead to false-positive identifications as a result of the multiple testing problem it does warrant further investigation of these identified proteins.

The individual measurements of the 11 proteins that exhibited statistically significant differences between the MG, RA and HC groups are summarized in the heat map shown in Fig. 1d along with Apolipoprotein A1 (APOA1) and the constant region of immunoglobulin heavy chain (IGHG2) as controls. In addition, the corresponding ANOVA F-statistic and p-values are also listed for each protein. The data reveals that fibrinogen-α (FGA), fibrinogen-β (FGB), fibrinogen-γ (FGG), keratin, type II cytoskeletal 78 (KRT78), ribonuclease 4 (RNASE4) and prothrombin (F2) are consistently in higher abundance in MG serum. Serum paraoxonase/arylesterase 1 (PON1), L-lactate dehydrogenase B (LDHB), and sex hormone-binding globulin (SHBG) are consistently high in RA sera, while complement C1q subcomponent subunit B (C1QB) and insulin-like growth factor-binding protein complex acid-labile subunit (IGFALS) are low in both MG and RA sera.

### High fibrinogen in MG sera

To narrow our focus, proteins consistently exhibiting the largest observed differences were selected for further investigation. As a control for the quantification by extracted ion intensities, the relative extracted ion intensities for a series of control are shown in Fig. 1e. The quantified data for the control proteins, including several immunoglobulins, apolipoproteins, albumin and C4b-binding protein alpha chain, reveal the spread in the quantified data for all three groups (MG, RA, and HC). For the proteins observed in differential abundance, all three fibrinogen subunits (FGA, FGB and FGG) exhibited the largest differential abundance in MG patient sera. As seen in Fig. 1e, the amounts of these fibrinogens were consistently higher in abundance by over an order of magnitude compared to controls (p < 0.01) and were thus selected for further biochemical validation.

### Validation of high residual fibrinogen in MG patient sera

**Figure 2:**
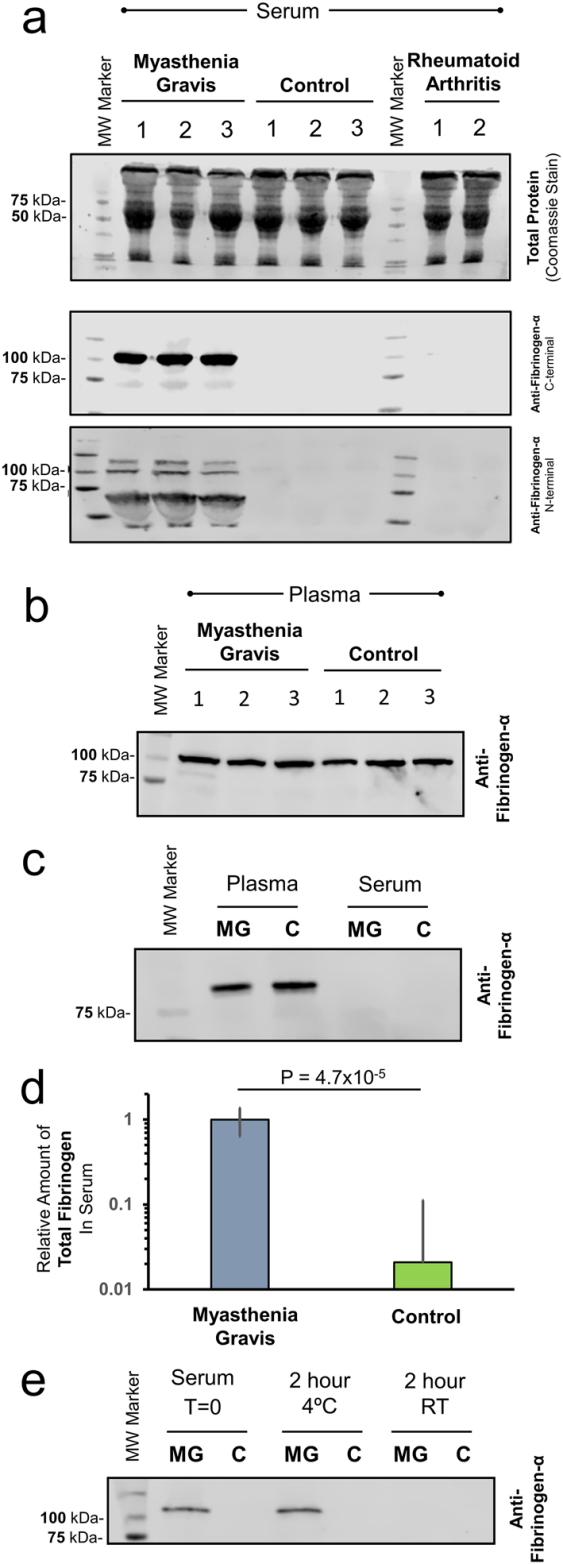
Immunochemistry Detection of Residual Fibrinogen in Serum. (**a**) (*Upper Blot*) Total protein loading of patient serum in an SDS-PAGE gel visualized by Coomassie staining. Analysis of the same protein loading of samples analyzed by Western blotting with anti-Fibrinogen-α antibodies derived against the **C-terminal** 750–850 residues (*Middle Blot)* or the **N-terminal** 21–320 residues (*Lower Blot*) of Fibrinogen-α. (**b**) Western blot analysis for Fibrinogen-α in plasma does not reveal observable differences between MG or control samples. (**c**) A comparative analysis of Fibrinogen-α in both plasma and serum samples from MG and control individuals reveals that the residual Fibrinogen-α observed in the sera of MG patients (A) is below the dynamic range of detection by Western blot analysis when compared to plasma Fibrinogen-α. (**d**) ELISA was used to quantify the difference in total fibrinogen in MG or control serum samples. Of note, in order to compare MG and control samples by ELISA, the MG samples were diluted prior to analysis to achieve a signal in the linear response range for the method. The resulting dilution factor is incorporated in the normalized values presented. P-value is for a two-tailed T-Test. (**e**) Stability of residual Fibrinogen-α after initial processing (T = 0) was investigated by Western blot analysis after incubating the serum sample for an additional two hours at either 4 °C or room temperature.

To validate our observations of high serum fibrinogen levels in MG patients, serum samples from MG, RA and HC individuals, were analyzed for Fibrinogen-α by immunoblotting (Fig. 2). To ensure equal protein loading for analysis, samples were normalized to total protein loading as visualized by Coomassie Blue staining (Fig. 2a *upper panel*). Immunoblotting for Fibrinogen-α (Fig. 2a *middle panel*) reveals a very high protein amounts in MG sera, but is essentially undetectable in either the RA or control samples. The epitope for this anti-Fibrinogen-α antibody is mapped to the amino acid residues 750–850, so this large differential detection of Fibrinogen-α was further validated by using an anti-Fibrinogen-α antibody derived against the residues 21–320 (Fig. 2a *lower panel*), that resulted in essentially the same results in high levels only being observed in sera from MG patients. This second antibody for the N-terminal region of fibrinogen did reveal altered apparent gel mobilities for Fibrinogen-α, suggesting that proteolytic processing is occurring that was not apparent by the analysis with the C-terminal specific antibody. Of note, the C-terminal specific antibody was used in all additional immunoblotting experiments.

This observed high abundance of fibrinogen is peculiar as to our knowledge there have been no previous reports of high fibrinogen in MG patients, nor is there any clotting disorder typically associated with the disease. As a result, we then investigated Fibrinogen-α amounts in the plasma of MG and controls by immunoblotting (Fig. 2b). The analysis revealed no significant difference in Fibrinogen-α in MG plasma when compared to a control sample. To investigate whether Fibrinogen-α was being removed during the collection and preparation of patient serum, both the plasma and serum of an MG patient and a control sample were analyzed simultaneously (Fig. 2c). What is observed is that when analyzing plasma, the Fibrinogen-α in serum is undetectable as a result of the limited dynamic range of Western blotting. Only when analyzing serum alone, are we able to observe a high level of residual Fibrinogen-α in the sera of MG patients (Fig. 2a).

As a result of the limited dynamic range of detection by immunoblotting, we conducted an ELISA assay for total fibrinogen. Due to the large differences in fibrinogen detected, the MG serum samples required dilution in order for them to be on the same linear range of detection for the assay when comparing to control samples. The resulting data from the ELISA is shown in Fig. 2d and like the proteomic analysis, reveals over an order of magnitude difference when comparing total fibrinogens in MG and control sera.

### Residual sera fibrinogen is stable at low temperature

To investigate the stability of the observed residual fibrinogen in the MG patient sera, the presence of fibrinogen was investigated upon prolonged incubations. Freshly thawed MG and control serum samples (T = 0) were further incubated for 2 h at either 4 °C or at ambient room temperature (~ 20 °C). Fibrinogen in the samples was then detected by immunoblotting with an anti- Fibrinogen-α antibody. As shown in Fig. 2e the residual fibrinogen persisted at low temperature but was no longer detectable upon incubating at room temperature, indicating the transient nature of the residual serum fibrinogen.

### Blinded analysis of residual serum fibrinogen identifies MG patients

Prior to investigating a large cohort of patients, a blinded study was initiated to verify whether the correlation of serum fibrinogen and MG held true with a new set of blinded samples.

**Figure 3:**
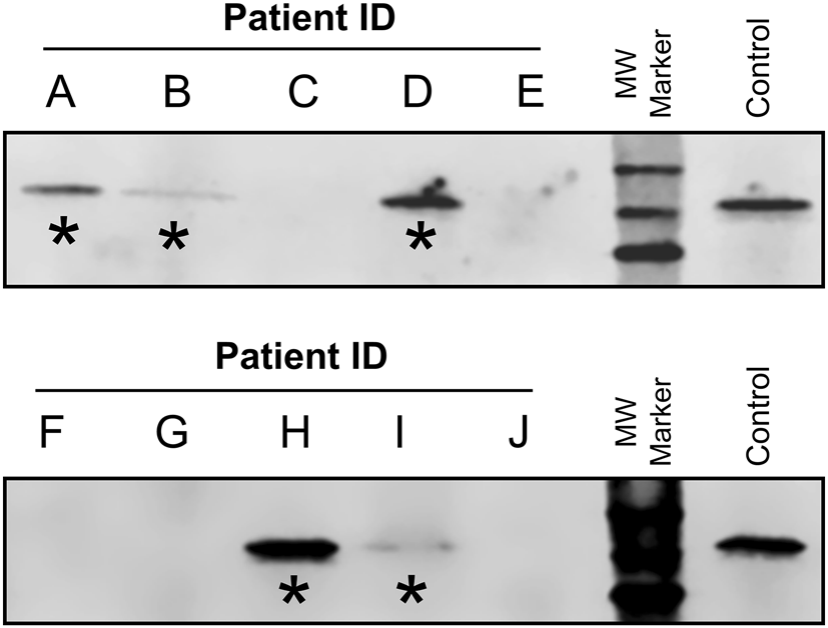
Blinded Investigation of MG Serum Samples. Ten blinded clinical serum samples from either controls or MG patients (A–J) were analyzed by Western blot analysis for residual Fibrinogen-α with serum from a MG patient used as a control. The serum samples A, B, D, H and I all exhibited detectable Fibrinogen-α upon analysis which exactly corresponded to the five samples from patients with MG (*).

A total of 10 blinded sera samples (A-J) were obtained from the MG clinic and analyzed for Fibrinogen-α by Western blotting. The resulting analysis revealed protein bands corresponding to Fibrinogen-α for samples A, B, D, H and I (Fig. 3). Unblinding of the clinical data revealed a 100% identification rate for MG patients from normal controls (patients C, E, F, G & J).

### Cohort analysis by targeted mass spectrometry for residual serum fibrinogen

With the promising findings of the initial proteomic study with regards to the correlation of fibrinogens (Fig. 1) and MG diagnosis along with the result of the blinded study with fibrinogen-α (Fig. 3) we then conducted a validation study on a larger patient cohort consisting of sera from 31 MG patients (The serotype of each of these patients was initially blinded and revealed after the analysis was complete: 27 anti-AChR antibody positive, one anti-MuSK antibody positive, three seronegative), 18 from RA patients and 30 controls. The demographic information and clinical profiles for this cohort are outlined in Table 1.

**Figure 4:**
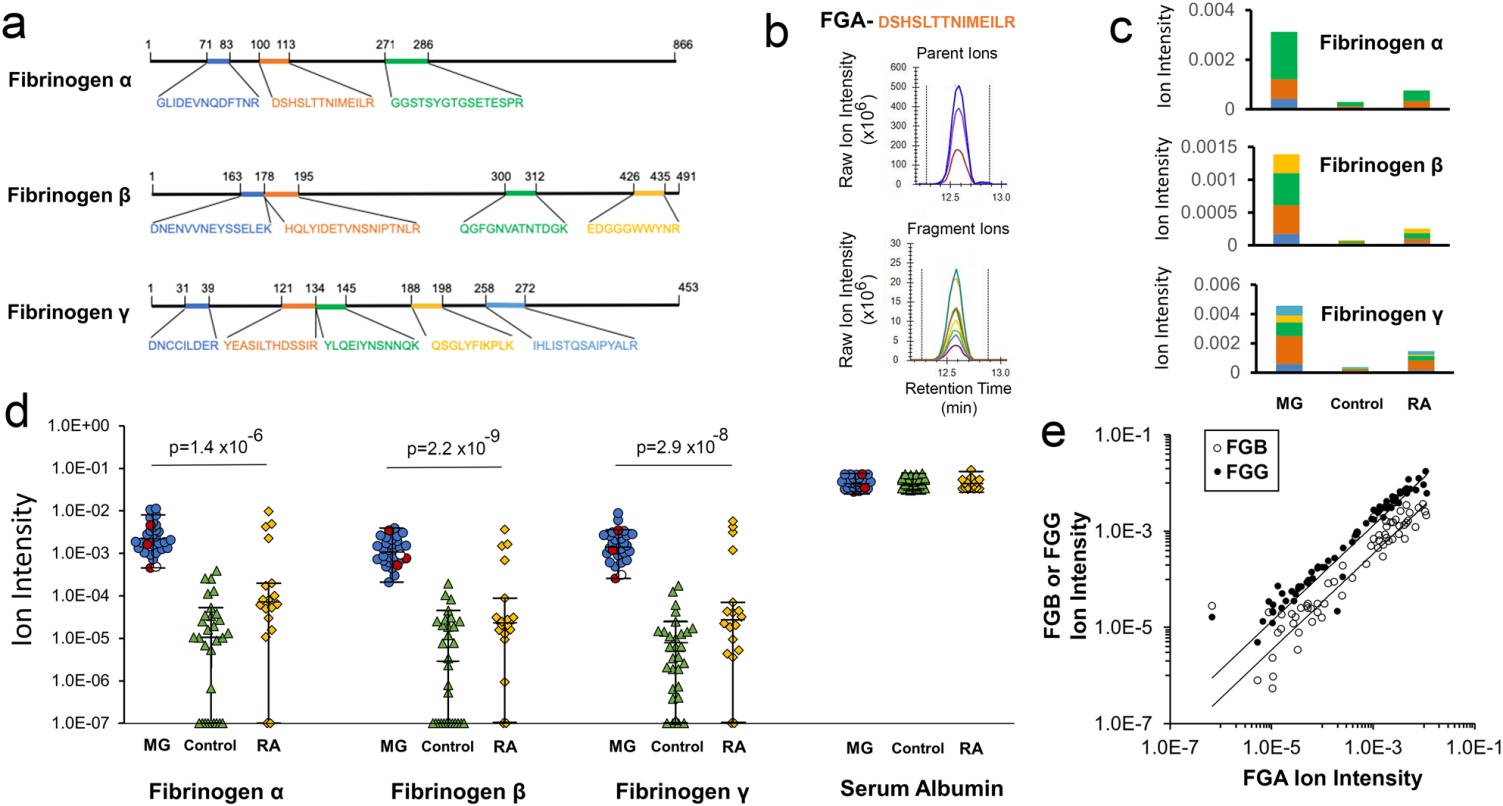
Targeted Proteomic Analysis of a Clinical Cohort. (**a**) For Parallel Reaction Monitoring (PRM), the indicated tryptic peptides from each fibrinogen were used for quantification by targeted LC–MS/MS analysis. (**b**) The extracted ion chromatograms (EICs) of co-eluting fragment ions aligned with the retention time of the precursor ion were quantified. Different coloured traces correspond to different peaks of the isotopic cluster or different fragment ion for the parent or fragment ions respectively. (**c**) Bar diagrams reveal the quantification of the sum of the EICs for the tryptic peptides derived from either fibrinogen-α, fibrinogen-β and fibrinogen-γ for single individuals with Myasthenia gravis (MG), Rheumatoid arthritis (RA) or control. Individual colour represents the EIC for the individual peptide quantified. (**d**) Beeswarm plots of the EICs quantified for the three fibrinogens and albumin as control for 31 MG patients, 18 RA patients and 30 controls. Individuals with undetectable levels of fibrinogen were assigned values at the threshold of detection. Within the MG cohort, of four patients negative for the anti-AChR autoantibody, one was positive for the anti-MuSK autoantibody (white circle) while three were double seronegative (red circles). (**e**) Linear regression analysis of fibrinogen-α, fibrinogen-β and fibrinogen-γ for all individuals reveals a high association for all the fibrinogens.

To circumvent the challenges of the limited dynamic range of detection observed for Western blot and ELISA assays, the serum samples were digested with trypsin and simultaneously analyzed for the tryptic peptides derived from fibrinogen-α, fibrinogen-β, fibrinogen-γ and serum albumin (as a control) by parallel reaction monitoring (PRM). PRM is a targeted mass spectrometry technique that results in reduced analytics variability than the label-free shotgun proteomic analysis^22^ used in our initial experiments identifying the fibrinogens. For PRM analysis of the 3 peptides for fibrinogen-α, 4 peptides fibrinogen-β, and 5 fibrinogen-γ targeted for quantification are listed in Fig. 4a. The four peptides for the serum albumin control are listed in Supplementary Table 4. For the present study, extracted ion chromatograms (EICs) of minimum five co-eluting fragment ions were measured for the quantification for each peptide, while being verified by aligning the retention time with the precursor ion for the given peptide to eliminate any possible mass spectrometry artifacts and quality assurance of the identified fragment ions. Samples raw data for the EIC of the fibrinogen-α derived DSHSLTTNIMEILR peptide is shown in Fig. 4b. The quantified abundance of each protein was the sum of EICs of corresponding peptide fragment ions normalized to serum albumin (Fig. 4c).

The quantification of fibrinogen-α, -β and -γ in the 79 serum samples by PRM analysis, shown in the Beeswarm plots in Fig. 4d, clearly reveals an essentially equally high abundance of all three fibrinogens in MG patient sera when contrasted to either control or RA serum samples. Of note, the sera from three seronegative and one anti-MuSK MG patients were indistinguishable from the other MG patients with regards to all three fibrinogens. While the majority of control and RA samples exhibited a significantly lower abundance of the fibrinogens, few individuals did reveal high fibrinogen levels. Within the dynamic range of detection of about five orders of magnitude with the PRM analysis, the median values for the three fibrinogens in MG samples were 28 to 54-fold higher than the RA samples and 250 to 4000-fold higher than the control samples. The MG sera revealed about a tenfold variation among individuals for the three fibrinogens, while the controls and RA sera exhibited about a 1000-fold variation. Nonetheless, one-way ANOVA analysis revealed high degrees of statistical confidence for fibrinogen-α, fibrinogen-β and fibrinogen-γ with p values of 1.4 × 10^–6^, 2.2 × 10^–9^ and 2.9 × 10^–8^, respectively.

With the similar patterns of outliers for all the fibrinogens (Fig. 4d), the correlations of fibrinogen-α, fibrinogen-β and fibrinogen-γ abundance in all patients was further investigated. Plotting fibrinogen-α abundance in an individual against either fibrinogen-β or fibrinogen-γ revealed significant multicollinearity among the proteins (Fig. 4e). This lack of fibrinogens acting as independent variables is not surprising as they are all subunits of the larger fibrinogen complex.

**Figure 5:**
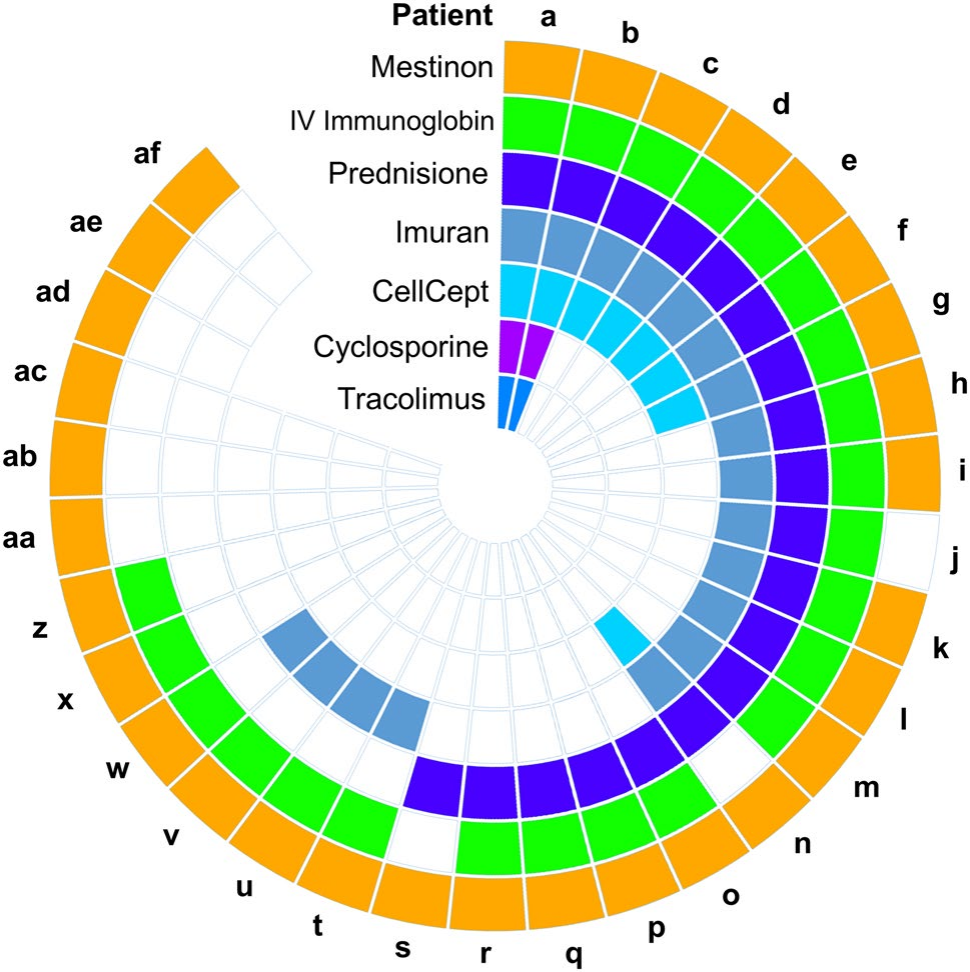
Prescription Drug Use of MG Patients. To investigate a possible association of prescribed drugs to MG patients with the observed high serum fibrinogen, the drug regime of the patient cohort was investigated as a potential variable. The sunburst plot reveals the prescription drug use of all MG patients (a–af) in the cohort. Comparison of prescriptions revealed no universal treatment that may have been a potential source of the universally high serum fibrinogen observed in these patients. (Final Graph was generated with Excel from Microsoft Office 365. Graphic was generated with Power Point from the same application package.)

We also considered the possibility that the observed residual fibrinogen could potentially be a treatment effect and not the result of MG. The available clinical records of the MG cohort were investigated with respect to the current and past drugs used by individual patients. The diversity in the drug regiments of these individuals (Fig. 5) and the occurrence of high residual fibrinogen in some controls and individuals with RA led us to conclude that these treatments are not the source of high residual fibrinogen.

### Residual serum fibrinogen for MG diagnosis

**Figure 6:**
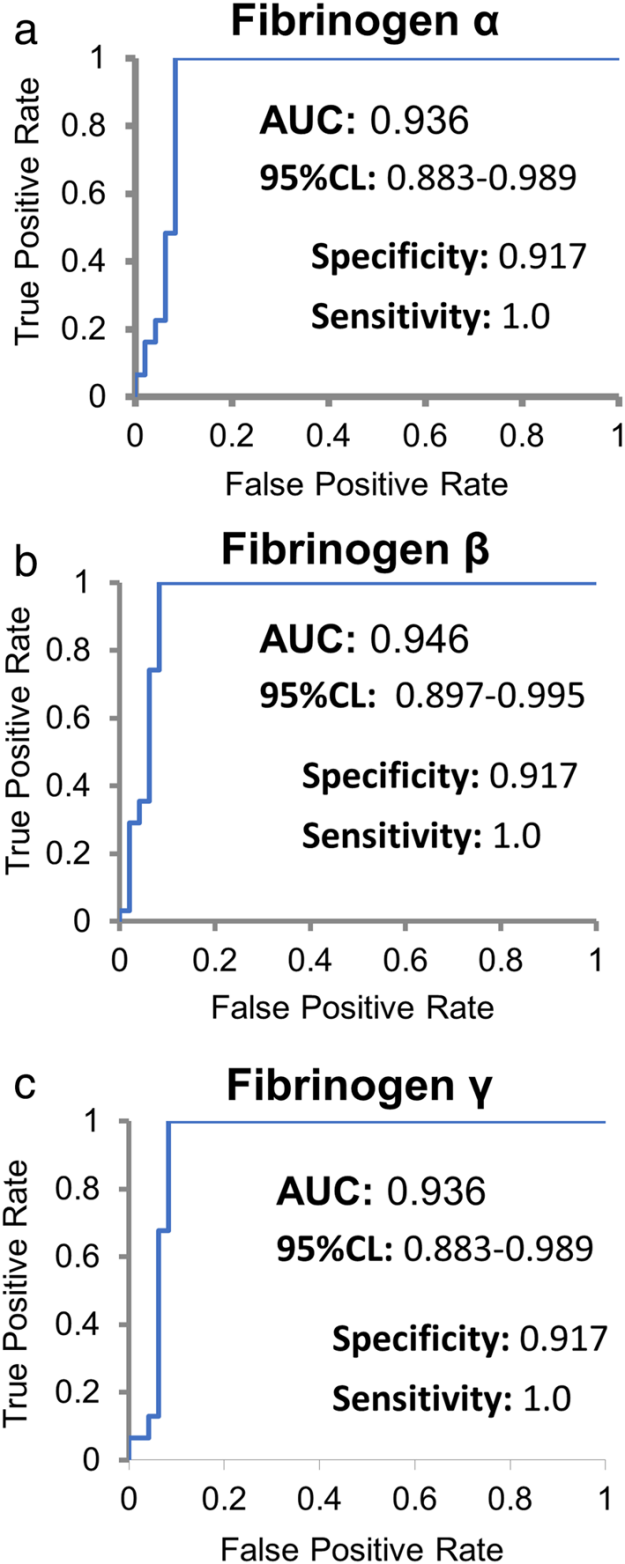
Receiver-Operator Curve (ROC) Analysis for High Serum Fibrinogen Proteins in MG. The results of the targeted proteomic analysis of Fibrinogen-α/β/γ of the patient cohort (Table 1) shown in Fig. 4 were analyzed for each Fibrinogen form indicated using ROCs comparing MG patients (n = 31) and the combined population (n = 48) of controls and RA patients. The areas under the curve and their 95% confidence intervals in addition to the specificities (True Positive Rate) and sensitivities (True Negative Rate) for each protein are provided in their respective inserts.

With the observed high statistically confident association between MG and residual serum fibrinogen, the data from the PRM analysis was applied to the receiver operator curve (ROC) analysis. For the ROC analysis, the data from the MG patients were compared to the combined pool of both RA and controls. Of note, we believe the comparison to the combined pool of RA and control samples is a better reflection of a true test scenario, but if the RA samples are excluded from the comparison, an AUC of 1.0 is calculated for MG patients versus controls. The resulting ROC analysis for fibrinogen-α, fibrinogen-β and fibrinogen-γ are individually shown in Fig. 6. ROC analysis was not performed combinatorically with the three fibrinogens as a result of the multicollinearity of these proteins (Fig. 4e).

## Discussion

Despite recent advances in the understanding and treatment of MG^1,27–29^, challenges remain with the timely diagnosis of this autoimmune disease as a result of its heterogeneous nature, non-specific symptoms and lack of availability of a universal biomarker^2,3^. A prior clinical study reported a greater than five-year delay in diagnosis of MG in 13% of patients^30^. Accurate diagnosis of MG remains challenging, more so in an emergency room setting, as MG patients may present with atypical symptoms mimicking other neurological conditions, particularly stroke, multiple sclerosis etc., that like MG, can also cause double vision and/or limb weakness. For molecular testing, the occurrence of the multiple serotypes and seronegative patients leads to limitations in the sensitivity of detection of MG with molecular markers.

To identify a potentially novel general serological marker for MG that may cover all isotypes, we conducted a three-way proteome profiling investigation of the sera of MG patients in comparison with both control individuals and those from a reference autoimmune disease, RA.

Our initial pilot study on a limited number of patients revealed potential protein candidates that exhibited statistically significant enrichment for each test group (Fig. 1) for which we believe are well representative of the test groups. For example, our data identified an increased abundance of the sex hormone-binding globulin (SHBG) in the sera of RA patients, a protein previously reported to exhibit a higher risk association for RA in women^31,32^. From our data, the proteins that exhibited the most striking degree of statistical confidence to be specifically associated with MG, were the three fibrinogen subunits (Fig. 1) that were then chosen for further investigation.

The finding of the α-, β-, and γ-subunits of fibrinogens was unexpected as there are no known associations of MG with any coagulopathy, which one may envision with the detection of unreacted fibrinogen in sera^33^. Further investigations revealed that the fibrinogens detected in the sera of MG patients were truly residual, as the comparisons with plasma revealed normal fibrinogen-α levels and the reduction in fibrinogen-α levels during the generation of sera appear normal when contrasted to plasma (Fig. 2b) as a result in the limited dynamic range of detection. While the majority of fibrinogen is removed upon the preparation of serum, very high residual amounts were constantly observed in MG patients in contrast to normal controls or RA patients. Quantitatively, the magnitude of the residual fibrinogen-α in MG sera was an order of magnitude higher in abundance as determined by mass spectrometry, Western blotting and ELISA (Figs. 1 and 2). While the observed high residual fibrinogen detected in sera prepared on ice, the fibrinogen was cleared upon incubation at ambient temperature (Fig. 2e), suggesting that this hypocoagulability was not likely to occur in vivo.

We investigated whether the high residual serum fibrinogen correlation to MG was a statistical anomaly or whether this association was repeatable in additional samples. Prior to investigating a larger patient cohort, a blinded study was conducted with randomized serum samples, where the detection of fibrinogen-α led to the 100% successful identification of MG patients (Fig. 3).

Based on the positive results of our blinded study, we analyzed a larger patient cohort by mass spectrometry using the quantitative PRM methods to quantify all three fibrinogens in sera (Fig. 4). These analyses confirmed a high specific association of serum fibrinogens with MG when contrasted to controls or RA samples, although the correlation was not absolute as a few samples in the control and RA groups also exhibited high residual fibrinogen levels, though of a much lower magnitude.

The strong correlation of serum fibrinogens with MG diagnosis observed by ROC analysis comparing MG patients to the combined group of both control and RA patient sera reveals high sensitivity (100% True Positive Rate (TPR)) and specificity (92% True Negative Rate (TNR)). We calculate an Accuracy of 95% and Likelihood Ratios (LR) of 12.00 and 0.00 for the positive and negative LRs, respectively. As a comparison, the diagnostic sensitivity and specificity of SFEMG, an advanced diagnostic technique restricted to only major academic centers, is 98% TPR and 70% TNR, respectively for the diagnosis of generalized MG^34^ and 79% TPR and 80% TNR, respectively for ocular MG^35^. In addition, the sensitivity and specificity within our sample set are higher than those for serological testing for the various autoantibodies. The comparative sensitivity of serum anti-AChR antibody for generalized and ocular MG diagnosis is at best about 92% TPR and 70% TNR, respectively^10,36^. Importantly, the samples from Class I patients in our set (Table 1) in addition to both the anti-MuSK and seronegative samples all exhibited high residual serum fibrinogens (Fig. 4).

To highlight the potential utility of the large magnitude of the observed differences in serum fibrinogens for diagnosis of MG, we arbitrarily reduced the cut-off for assignment for high fibrinogens to ten-fold below that for the lowest observed case for a MG sample for ROC analysis. With the reduced cut-off, the accuracy of predicting MG only drops to 75.9% (While the sensitivity remains at 100%). In contrast, the quantification of inflammatory proteins^12^ and metabolites^16^ identified a twofold difference between MG and the control groups.

The observation of high serum fibrinogen interfering with analysis in cases not attributed to dysglobulinemia, have been reported, but its origin was unknown^37^. We queried whether the uniform observation of high serum fibrinogen may have been linked to the treatment of MG, as mentioned in the results. This does not appear to be the case due to the considerable diversity of pharmacological treatment of the MG cohort in addition to the rare detection of high fibrinogen in some non-MG individuals. Potential cross-reactivity of auto-antibodies to fibrinogen may protect fibrinogen from activation, but this seems unlikely as it appears to occur with all serotypes. Interestingly, a previous proteome-wide screening for cross-reactivity of autoantibodies in MG patients failed to report fibrinogen as a potential target^38^. Nonetheless, other factors may bind fibrinogen and preclude its conversion to fibrin. Further investigation will be required to elucidate the biochemical mechanisms resulting in the elevated residual serum fibrinogen levels.

The underlying pathophysiology of highly elevated residual serum fibrinogen in MG is unclear. Fibrinogen is a plasma protein that is synthesized in liver and is involved in the blood clotting cascade. Fibrinogen is upregulated by inflammatory mediators and elevated levels have been reported during the acute phase of various autoimmune/inflammatory diseases including RA, inflammatory bowel disease, vasculitis, multiple sclerosis and chronic obstructive pulmonary diseases etc^39,40^. In MG patients fibrinogen levels have not been systematically studied and elevation of serum fibrinogen levels in MG has not been observed previously except in one patient, recently^41^. MG is an antibody mediated disease that leads to destruction of the post-synaptic membrane through release of inflammatory mediators and complement activation. Treatment can reduce the serum AChR antibody levels, but the antibodies can be detected even in well controlled MG^42^ and possibly, continue to attack their target antigens at the NMJ as several cytokines are elevated in the sera of MG patients as compared to controls^11^. The observation that fibrinogen levels were increased in all types of MG in our cohort, regardless of the severity of disease, suggests an ongoing subclinical inflammatory process at the NMJ.

In summary, we have identified high levels of residual fibrinogen in all patients diagnosed with MG. This discovery was enabled by our combination of unbiased shotgun proteomics, Western blotting, ELISA and targeted mass spectrometry of unaltered serum. We found the presence of high serum fibrinogen levels to be highly sensitive to MG with specificities that appear to be potentially superior to current clinical testing methods. Additionally, high residual serum fibrinogen is also detected in all serotypes (within the scope of our current limited sample size) as well in the Class I patients (a purely ocular form of MG), indicating that residual serum testing may be more advantageous than current serological tests. We anticipate our findings to provide the foundation for further studies and potentially the development of a routine approach for early MG diagnosis.

### Supplementary Information

Supplementary Tables.

Supplementary Figures.

## Data Availability

The raw mass spectrometry data files will be available on ProteomeXchange Consortium via PRIDE under project title “Serum Proteomic Profiling of Myasthenia gravis” and project accession—PXD019633. The data will remain confidential until published.
