# Supplementary material for: Residual serum fibrinogen as a universal biomarker for all serotypes of Myasthenia gravis
Source: Sci Rep. 2023 Dec 1;13:21229. doi: 10.1038/s41598-023-47559-x (PMC10692328; doi:10.1038/s41598-023-47559-x)
Supplement: Supplementary file 2 — Supplementary Figures. [file 41598_2023_47559_MOESM2_ESM.pdf]

Residual Serum Fibrinogen as a Universal Biomarker for all Serotypes of Myasthenia Gravis

Faraz S. Hussain[1], Ramanaguru S. Piragasam[2], Hassan Sarker[2], Derrick Blackmore[1], Elaine Yacyshyn[3], Carlos Fernandez-Patron[2], Richard P. Fahlman[2,*] & Zaeem A. Siddiqi[1,*].

[1] Division of Neurology, Department of Medicine, Faculty of Medicine & Dentistry, University of Alberta.
[2] Department of Biochemistry, Faculty of Medicine & Dentistry, University of Alberta.
[3] Division of Rheumatology, Department of Medicine, Faculty of Medicine & Dentistry, University of Alberta.

*To whom correspondence should be addressed: zsiddiqi@ualberta.ca or rfahlman@ualberta.ca

Supplemental Data

Supplemental Information in the forms of Supplemental Tables are attached in Microsoft office Excel. Supplemental Table 1: Raw data – extracted ion intensities; Supplemental Table 2: Corrected data – normalized data; Supplemental Table 3: ANOVA – analysis of variance; Supplemental Table 4: PRM ion lists – parallel reaction monitoring peptide and transition ion list; Supplemental Table 5: PRM data – raw parallel reaction monitoring data.

Supplementary Data: Uncropped Blot Images for Review Process

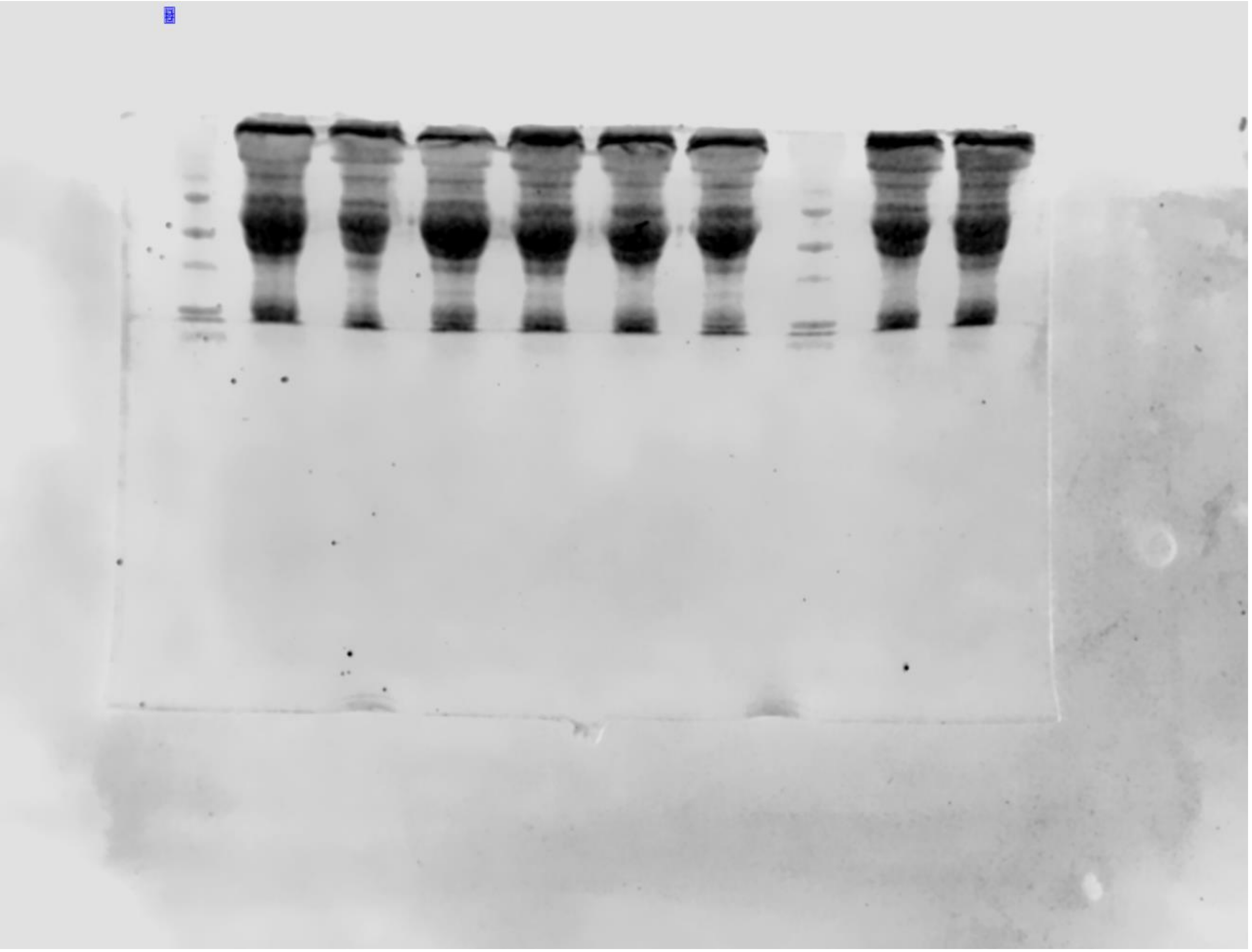

Supplementary Figure S1: This is the uncropped image for the Figure 2a (*Upper Blot)*, representing western blot analysis for total protein loading

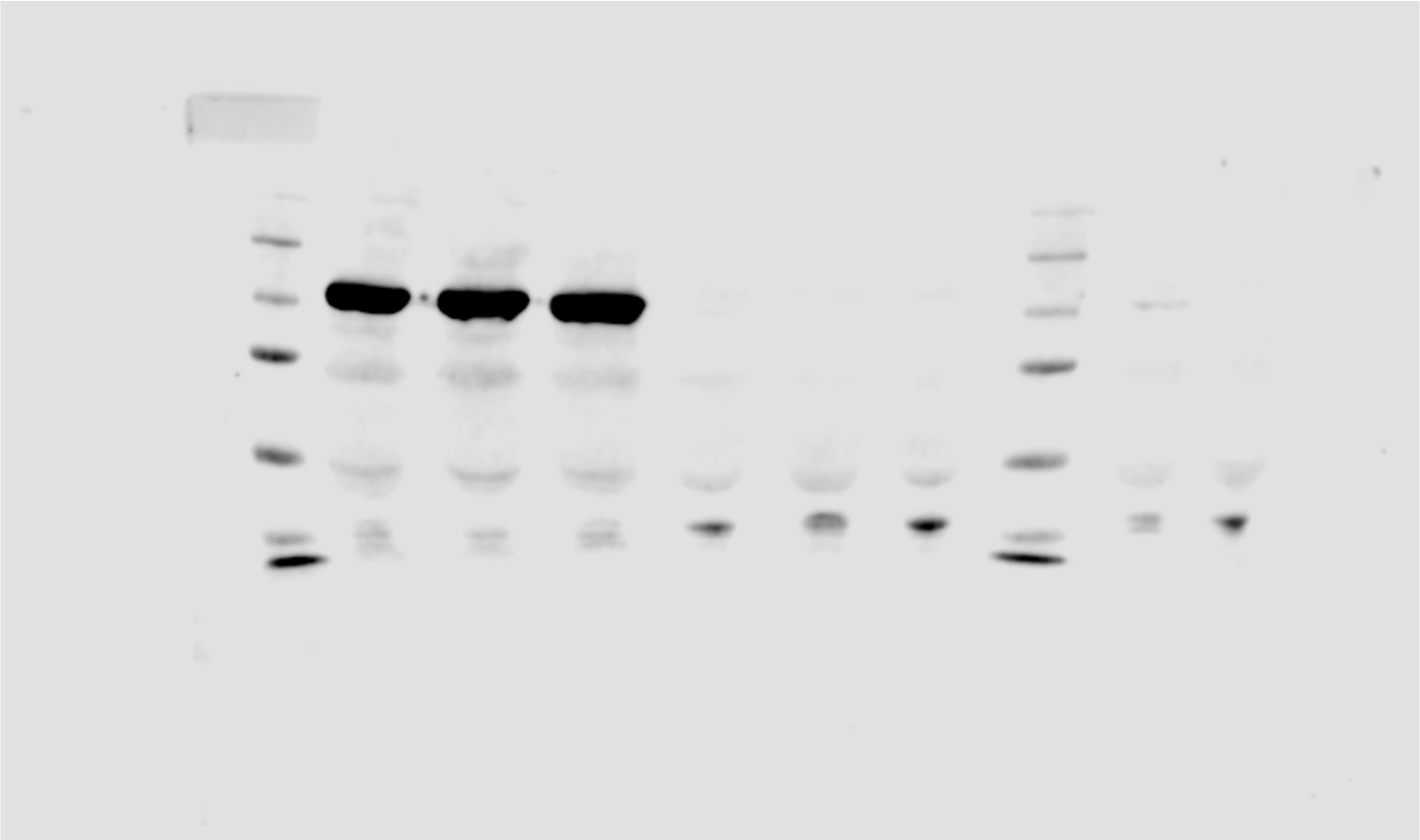

Supplementary Figure S2: This is the uncropped image for the Figure 2a (*Middle Blot*), representing western blot analysis for Fibrinogen-α C-terminal (specific to 750-850 residues)

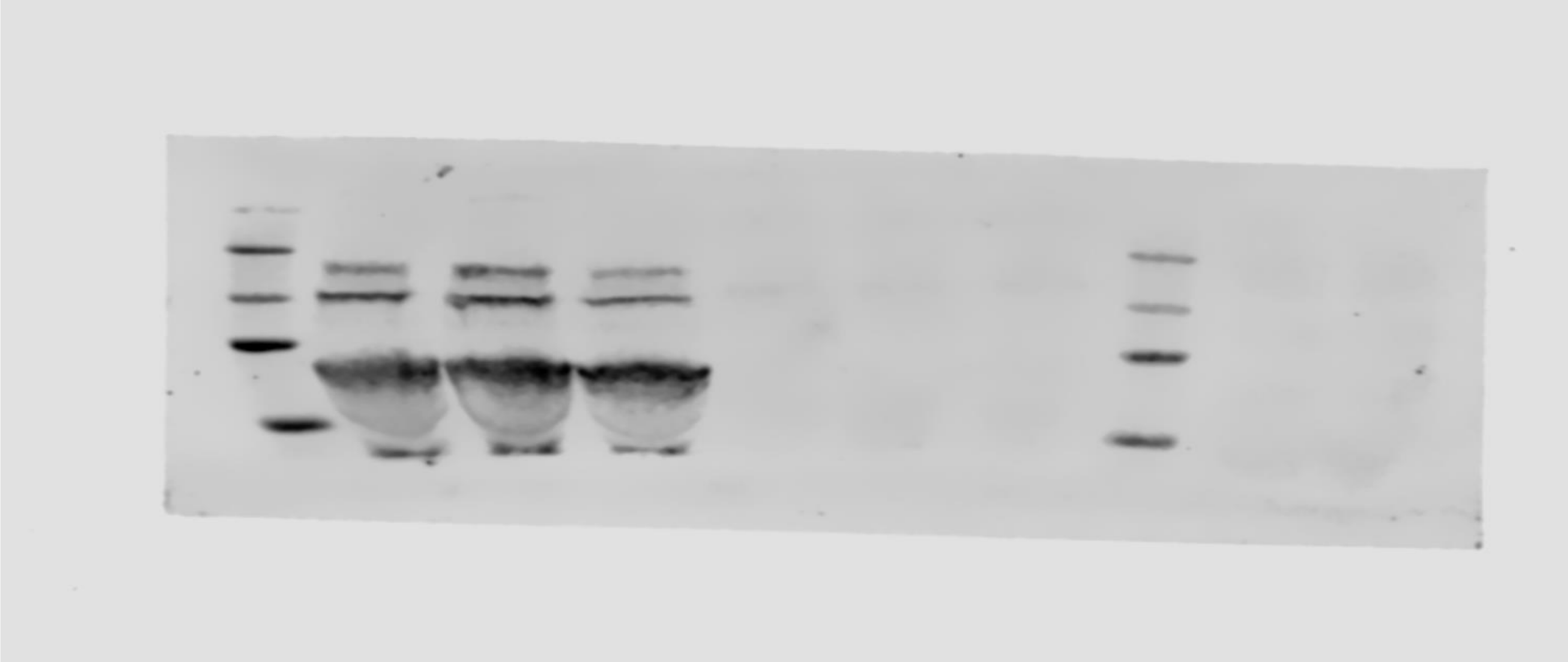

Supplementary Figure S3: This is the uncropped image for the Figure 2a (*Lower Blot*), representing western blot analysis for Fibrinogen-α N-terminal (specific to 21-320 residues)

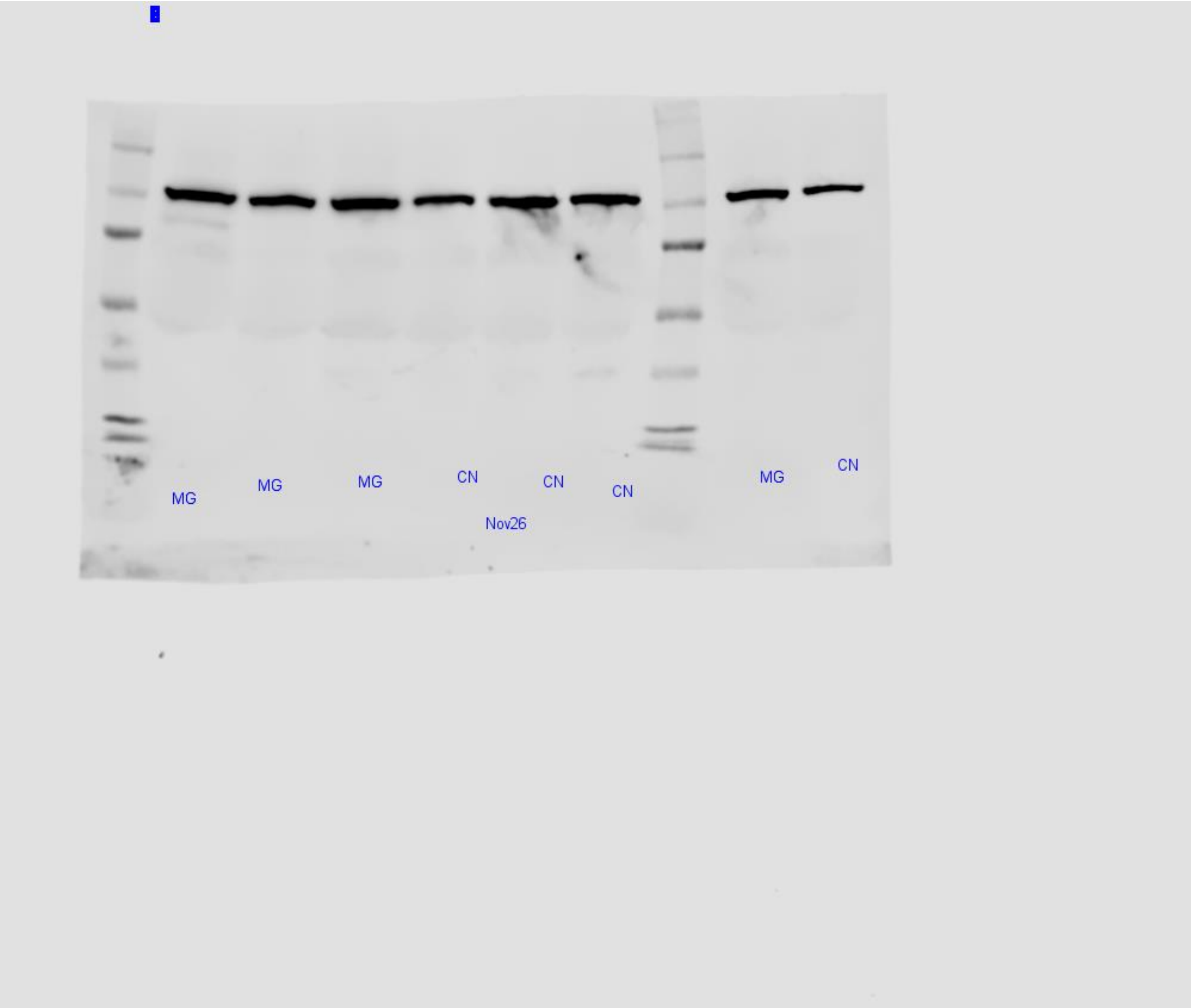

Supplementary Figure S3: This is the uncropped image for the Figure 2b, representing western blot analysis for Fibrinogen-α in plasma

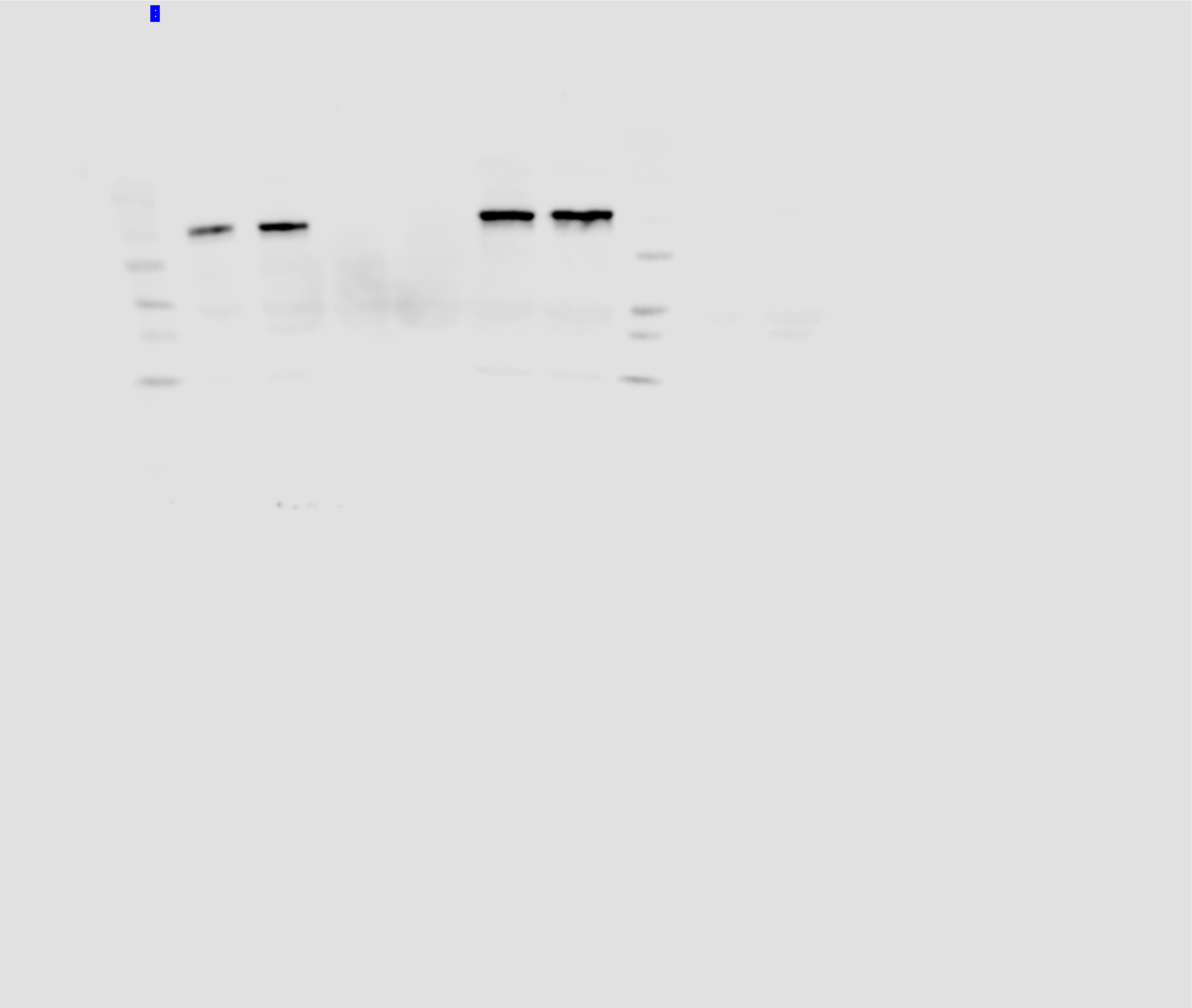

Supplementary Figure S4: This is the uncropped image for the Figure 2c, a comparative analysis of Fibrinogen-α in both plasma and serum

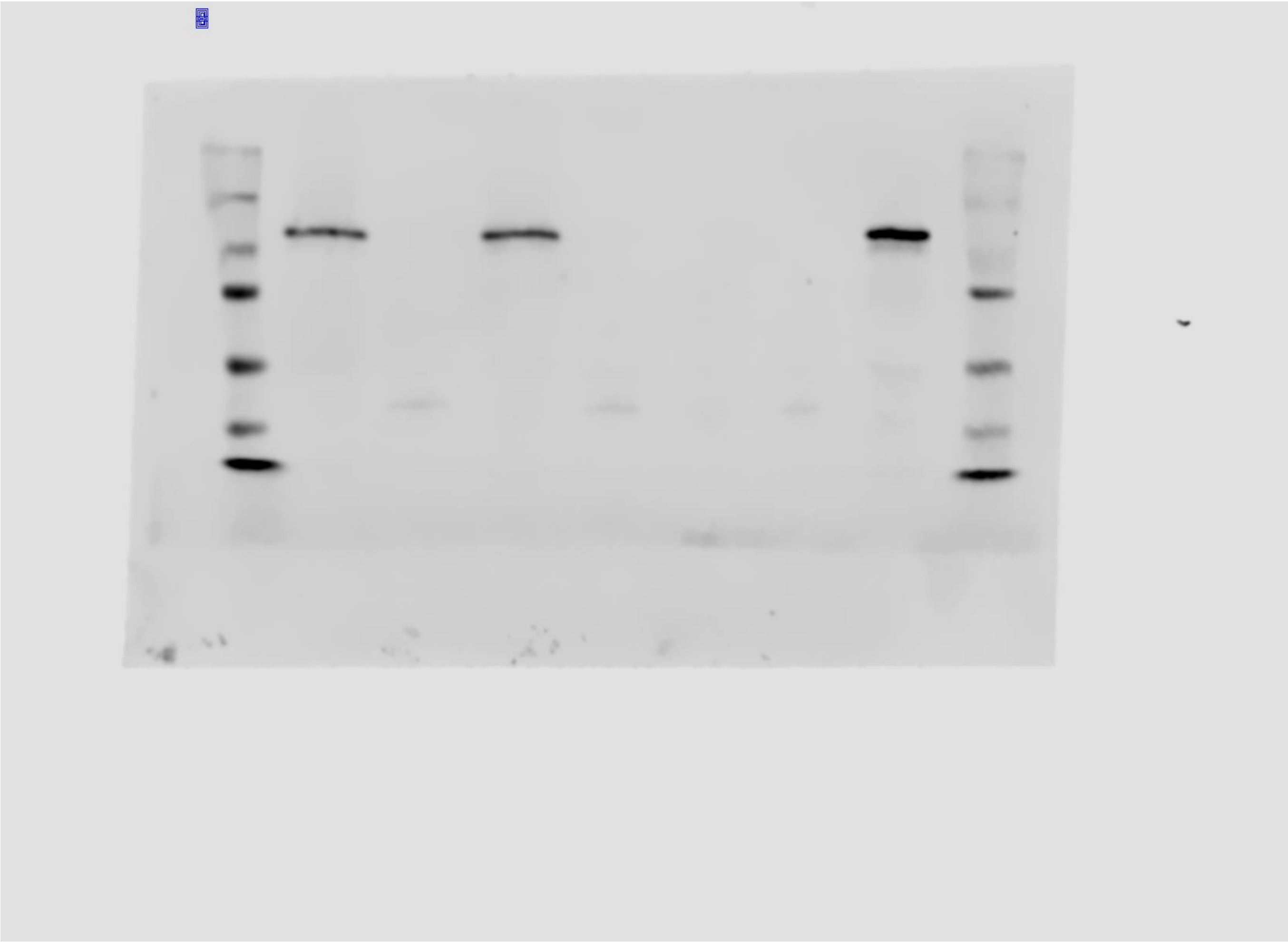

Supplementary Figure S5: This is the uncropped image Figure 2e, representing stability of residual Fibrinogen-α after initial processing

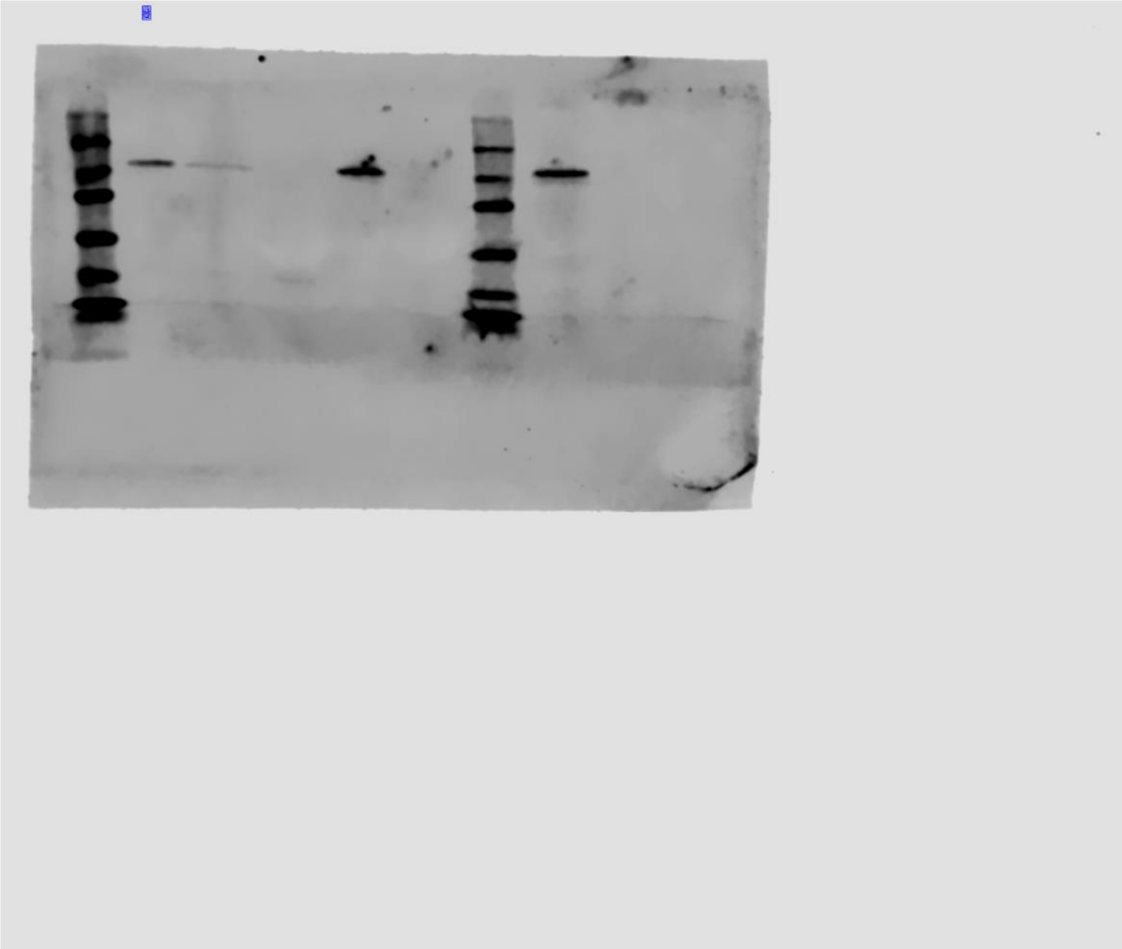

Supplementary Figure S6: This is the uncropped image Figure 3 (Upper Blot), representing the blinded Investigation of MG serum samples

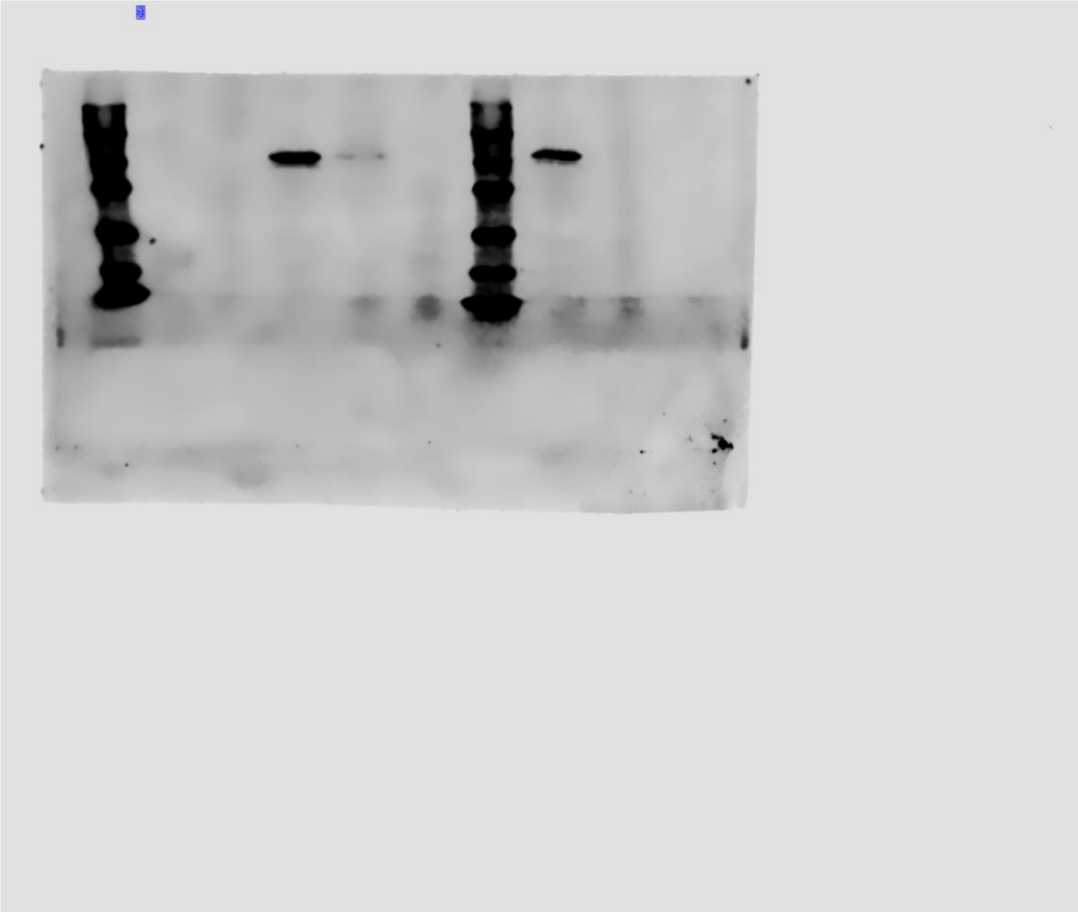

Supplementary Figure S7: This is the uncropped image Figure 3 (Lower Blot), representing the blinded Investigation of MG serum samples
